# Editorial: From the heart of the athlete to athlete's heart to cardiopathy: approaches and challenges in risk management in cardiovascular sports medicine

**DOI:** 10.3389/fcvm.2026.1953095

**Published:** 2026-08-13

**Authors:** Clotilde Castaldo, Franca Di Meglio, Daria Nurzynska

**Affiliations:** 1 Department of Public Health, University of Naples “Federico II”, Naples, Italy; 2 Department of Medicine, Surgery and Dentistry, University of Salerno, Baronissi, Italy

**Keywords:** athlete's heart, cardiomyopathy, cardiovascular risk, risk assessment, sports medicine

Regular athletic training induces structural and functional adaptations in the heart, a phenomenon collectively referred to as “athlete's heart.” Over the past two decades, extensive research has clarified the typical patterns of cardiac remodeling in athletes. However, distinguishing some forms of physiological adaptation from inherited or acquired cardiac pathologies remains a diagnostic challenge.

The specific type of adaptation depends heavily on the activity (1), with resistance and power training characterized by high-intensity, static-isometric exertion in anaerobic sports, associated with a progressive increase in pressure overload that can lead to concentric hypertrophy, and endurance training common in aerobic sports, in which dynamic exercise reduces total peripheral resistance and enhances venous return, creating a volume overload that can result in eccentric hypertrophy, associated with decreased end-systolic volume, increased stroke volume, and electrical adaptations such as sinus bradycardia. Based on the above features, diagnostic imaging with functional measurements allows clinicians to identify a broad spectrum of heart conditions in athletes with great accuracy**.** However, understanding the continuum from normal physiological adaptation to pathological cardiomyopathy reflects a broader evolution in sports cardiology, which acknowledges that regular physical activity is among the most effective interventions for preventing and treating cardiovascular disease and thus aims at distinguishing between “safe” and “problematic” in order to support sport participation rather than simply enforcing medical restrictions (2). This Research Topic highlights the modern approach, which is based on diagnostic evidence, personalized risk management and shared decision-making (3).

The cardiovascular response to exercise is one of the most remarkable examples of biological adaptation. Regular training produces characteristic structural and functional cardiovascular remodeling that enables the cardiovascular system to meet increased physiological demands during exercise (4). Not surprisingly, the concept of the athlete's heart has become a key topic in sports medicine. Indeed, as participation in sport continues to increase across different age groups, disciplines, and competition levels, clinicians face a major challenge: distinguishing physiological adaptation from pre-existent or early cardiovascular disease. The six studies included in this Research Topic demonstrate that exercise-induced cardiovascular adaptation is not simply normal or abnormal. Instead, it represents a continuum influenced and shaped by training volume, age, sex, genetics, and individual traits. Together, these contributions support a modern approach based on accurate assessment, consistent follow-up, and personalized clinical decision-making.

As reminded earlier, cardiac remodeling is dynamic and athlete's heart develops according to the specific demands of each sport and the volume of training performed over time. Distinguishing physiological adaptations from pathological changes remains one of the main challenges in sports cardiology. A study by Polito et al. in elite futsal players showed that long-term, high-intensity intermittent training leads to progressive cardiac remodeling, including increased ventricular wall thickness and atrial enlargement, while keeping systolic function intact. These results highlight the importance of interpreting cardiac morphology and cardiac imaging in relation to training history, sport discipline, and changes over time. Especially since regular endurance training improves endothelial function and reduces arterial stiffness, demonstrating early vascular benefits, even when structural changes are not yet evident in the short term, as demonstrated by Bian et al. in their meta-analysis of randomized clinical trials on the effects of continuous aerobic exercise on atherosclerosis-related vascular indices in overweight and obese adults. Together, these findings show that the cardiovascular benefits of exercise develop gradually and involve several biological mechanisms.

Endurance sports present additional diagnostic challenges because intense physiological stress can produce extensive structural, functional, and electrical remodeling, accompanied by temporary changes that overlap with pathological findings and resemble cardiovascular disease (4). In the study by Lasocka-Koriat et al. in amateur marathon runners, extreme physical strain caused transient structural and functional changes, as assessed by echocardiography, as well as biochemical alterations, with Galectin-3 increase correlating with reversible functional decline. Interestingly, different responses were observed in male and female athletes. These findings show that cardiovascular responses to intense exercise are dynamic and reversible, while also emphasizing the need to identify athletes who require further clinical evaluation.

As athletes grow older, cardiovascular screening becomes more complex and challenging, as long-term training adaptations overlap with age-related changes and a higher risk of acquired heart disease. Re-evaluating ECG criteria in master (over 35 years-of-age) competitive athletes highlights the importance of refining screening tools to maintain an appropriate balance between sensitivity and specificity in all age groups. The retrospective study by Halasz et al. showed that the Seattle criteria demonstrated higher overall accuracy, with significantly better discriminative performance than the International criteria, and a lower false-positive rate compared with the ESC criteria for diagnosis of conditions at risk of sudden cardiac death.

Hypertrophic cardiomyopathy has traditionally led to major restrictions in competitive sports participation because of concerns about ventricular arrhythmias and sudden cardiac death. However, recent evidence supports a more personalized approach. The meta-analysis by Borrelli et al., included in this Research Topic, showed that structured exercise in carefully selected individuals with hypertrophic cardiomyopathy does not increase the risk of major adverse cardiovascular events and improves cardiorespiratory fitness. These cases highlight the value of multidisciplinary evaluation involving sports cardiologists, geneticists, imaging specialists, and long-term follow-up. This paradigm shift is reflected in updated international guidelines, which now recognize that universal restriction from vigorous exercise is not warranted for most patients with hypertrophic cardiomyopathy (5, 6). Furthermore, the cases of elite female athletes carrying pathogenic variants associated with Duchenne muscular dystrophy, analyzed by Wernhart et al., illustrate the challenges of evaluating athletes with a possible inherited risk of myocardial disease. Female carriers may have no clinical symptoms while still being at risk of myocardial involvement, raising important questions about the interaction between genetic predisposition and long-term exposure to high-intensity exercise.

Overall, the studies included in this Research Topic show that the future of sports cardiology goes beyond classifying athletes as either healthy or diseased. Exercise-induced cardiovascular remodeling occurs along a continuum and its correct interpretation requires the integration of clinical history, imaging, biomarkers, and genetics. Future developments in cardiovascular sports medicine will increasingly rely on precision medicine to identify individual risk while avoiding unnecessary restrictions on physical activity. Integrating advances in multimodality imaging, molecular biomarkers, and genomic medicine is expected to improve the ability to distinguish physiological adaptation from cardiovascular disease and to support more personalized exercise recommendations.

## References

[1] European Association of Cardiovascular Imaging. The athlete’s heart. EACVI Multimodality Imaging Toolbox on Cardiomyopathies and the Athlete’s Heart. European Society of Cardiology. Available online at: https://www.escardio.org/education/resources/imaging-toolboxes/cardiomyopathies-and-the-athletes-heart/the-athletes-heart/ (Accessed July 27, 2026).

[2] Pelliccia A Sharma S Gati S Bäck M Börjesson M Caselli S. 2020 ESC guidelines on sports cardiology and exercise in patients with cardiovascular disease: the task force on sports cardiology and exercise in patients with cardiovascular disease of the European Society of Cardiology (ESC). Eur Heart J. (2021) 42:17–96. 10.1093/eurheartj/ehaa605 32860412

[3] Isath A Koziol KJ Martinez MW Garber CE Martinez MN Emery MS. Exercise and cardiovascular health: a state-of-the-art review. Prog Cardiovasc Dis. (2023) 79:44–52. 10.1016/j.pcad.2023.04.008 37120119

[4] Martinez MW Kim JH Shah AB Phelan D Emery MS Wasfy MM. Exercise-induced cardiovascular adaptations and approach to exercise and cardiovascular disease: jACC state-of-the-art review. J Am Coll Cardiol. (2021) 78:1453–70. 10.1016/j.jacc.2021.08.003 34593128

[5] Writing Committee Members, Ommen SR Ho CY Asif IM Balaji S Burke MA. 2024 AHA/ACC/AMSSM/HRS/PACES/SCMR guideline for the management of hypertrophic cardiomyopathy: a report of the American Heart Association/American College of Cardiology joint committee on clinical practice guidelines. J Am Coll Cardiol. (2024) 83:2324–405. 10.1016/j.jacc.2024.02.014 38727647

[6] Arbelo E Protonotarios A Gimeno JR Arbustini E Barriales-Villa R Basso C. 2023 ESC guidelines for the management of cardiomyopathies. Eur Heart J. (2023) 44:3503–626. 10.1093/eurheartj/ehad194 37622657

